# Purpura Fulminans and Group B Streptococcal Sepsis in an Extremely Low Birth Weight Premature Infant

**DOI:** 10.1155/crdi/2925403

**Published:** 2025-12-22

**Authors:** Surasak Puvabanditsin, Aline Sandouk, Ian Lee, Mannan Shah, Amrryn Halari, Su Young Park, Rajeev Mehta

**Affiliations:** ^1^ Department of Pediatrics, Rutgers Robert Wood Johnson Medical School, New Brunswick, New Jersey, USA, rutgers.edu

**Keywords:** extremely low birth weight, Group B *Streptococcus*, premature infant, purpura fulminans, sepsis

## Abstract

Purpura fulminans (PF) is an ominous, rapidly progressive cutaneous manifestation of intravascular thrombosis and hemorrhagic infarctions typically mediated by coagulopathy or bacterial infection. Neonatal PF is a rarely reported but potentially disabling disorder associated with a high mortality rate and severe long‐term morbidity in survivors. We report an extremely low birth weight premature infant with PF associated with late‐onset Group B streptococcal sepsis. The infant survived but suffered a severe brain injury and autoamputation of all four extremities. We present this case and a review of the literature.

## 1. Introduction

Purpura fulminans (PF) is a destructive disorder characterized by symmetrical thrombotic and hemorrhagic necrosis of the skin and soft tissues. It is a form of consumptive coagulopathy and, in experimental animals, resembles a widespread Shwartzman reaction brought on by endotoxemia [1,2]. Vascular endothelial cells are damaged by endotoxins or immune complexes, the coagulation system gets activated and intravascular thrombosis and small‐vessel hemorrhages occur, which progress to consumptive coagulopathy [3]. PF in neonates has been linked to *Staphylococcus aureus*, *Neisseria meningitidis*, *Escherichia coli*, *Enterobacter* species, *Pseudomonas* species, and *Streptococcus agalactiae*, also known as Group B *Streptococcus* (GBS) [4,5]. Only a few cases of infectious PF in neonates have been reported. This is a rare event in the neonate in the era of the scarce incident of late‐onset GBS sepsis. We describe the onset and development of PF in a 108‐day‐old, extremely low birth weight female with GBS sepsis, complicated by a thrombotic process involving severe neurologic injury and necrosis of all limbs. This article aims to detail our experience with the evolution of PF, review the existing literature, and contribute a new association between PF and late‐onset neonatal GBS infection.

## 2. Case Presentation

**Figure 3:**
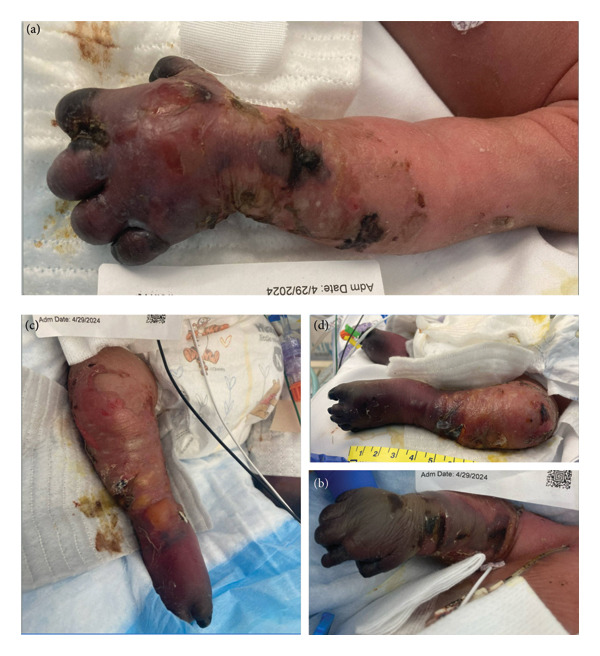
Photos taken 14 days after the onset of sepsis showed progressive necrosis of all extremities and autoamputation of fingers (a, b) and toes (c, d).

This is a female neonate conceived by in vitro fertilization who was born at 23 weeks of gestation to a 36‐year‐old gravida 2 para 0111 mother by vaginal delivery. Apgar scores were 5 and 8 at 1 and 5 min, respectively. Prenatal care was complicated with preterm premature rupture of membranes at 22 weeks and 4 days of gestation. Cerclage was placed. The mother was treated with levothyroxine for subclinical hypothyroidism. The family history was unremarkable. Birth weight was 450 g (< 10^th^ percentile), length was 28 cm (15^th^ percentile), and head circumference was 19 cm (10^th^ percentile). The physical exam at birth showed a hypotonic, inactive, small‐for‐gestational‐age infant without dysmorphic features in respiratory distress, requiring multiple resuscitative measures. She was admitted to the NICU and placed on nasal CPAP. The postnatal course was complicated by spontaneous intestinal perforation (SIP) at day of life (DOL) 13, and no evidence of clinical necrotizing enterocolitis (NEC) was noted. Ampicillin, gentamicin, and metronidazole were administered, and a peritoneal Penrose drain was placed on the right lower quadrant of the abdomen for 7 days. Enteral feeding was resumed 2 weeks after the SIP event, and full enteral feeding was achieved after 4 weeks. The infant developed bilateral retinopathy of prematurity (Stage II, Zone II, plus disease) at DOL 89, requiring laser treatment. She required noninvasive respiratory support after birth from DOL 56 onward. She also tolerated full enteral feeding. By DOL 108, her weight was 1630 g, and she was receiving 2 L per minute of 21% oxygen (room air) via nasal cannula when she developed brief desaturation episodes, and subsequently became apneic, pale, hypothermic, and lethargic. She was orally intubated and placed on mechanical ventilation. Blood culture and complete blood counts (CBC) were obtained. Empiric treatment with ampicillin, ceftazidime, and metronidazole was initiated. Intravenous dopamine was started for hypotension. CBC showed a white blood cell (WBC) count of 1230 cells per microliter, Hb of 10 g/dL, Hct of 33%, with a differential of 19% PMNs, 16% Bands, 49% lymphocytes, and 7% monocytes. C‐Reactive protein was 10 mg/dL. Blood culture grew penicillin‐sensitive GBS. She was started on anticonvulsant medications on DOL 109 for new‐onset seizures (tonic‐clonic movements of extremities), which was confirmed with video electroencephalography (EEG). After 18 h of antibiotic administration, ecchymosis was noted on all distal parts of her extremities. Her hemoglobin dropped, and she received a packed red blood cell transfusion. A coagulation study showed: platelets at 5000 per microliter, prothrombin time (PT) at 25 s (s) (normal: 10–13 s), partial thromboplastin time (PTT) at 69 s (normal: 20–31 s), international normalized ratio (INR) at 2.2, fibrinogen at 119 mg/dL (normal: 180–350 mg/dL), and D‐dimer at 62,632 μg/L (normal: 63–246 μg/L), suggestive of disseminated intravascular coagulation (DIC) with PF. Over the following days, the ecchymosis and ischemic skin lesions became more extensive (Figures 1, 2, 3, 4). She received multiple platelet and fresh frozen plasma transfusions over the next few days of the event. Her respiratory status was also complicated with respiratory failure and persistent pulmonary hypertension requiring inhaled nitric oxide and high‐frequency oscillatory ventilation, requiring 4 weeks of mechanical ventilation. She developed generalized edema necessitating diuretic therapy. Despite aggressive treatment with transfusion of blood products and antibiotics (ampicillin and cephalosporin), the ischemic lesions continued to worsen. Surgical consultation recommended deferring intervention until clearer demarcation of the evolving peripheral necrosis was observed. Surgical amputation of both forearms and legs was performed at DOL 155 (Figure 5). Cranial ultrasound at DOL 109 showed multiple intraparenchymal echogenic areas representing hemorrhage and infarction; subsequent imaging showed progressive ventriculomegaly and encephalomalacia (Figures 6 and 7). She developed severe hydrocephalus and encephalomalacia, prompting a ventriculoperitoneal shunt placement at DOL 192. A gastrostomy tube was placed to facilitate her feeding problem. At DOL 231, she was discharged to an inpatient rehabilitation facility for comprehensive care and management.

Figure 1Photos taken 24 h after the onset of symptoms of sepsis showed ecchymoses of the hands, feet, legs, and forearms (a–c). Several hours later, PF became evident with clearly demarcated cutaneous necrosis involving all distal extremities (d, e).

**Figure:**
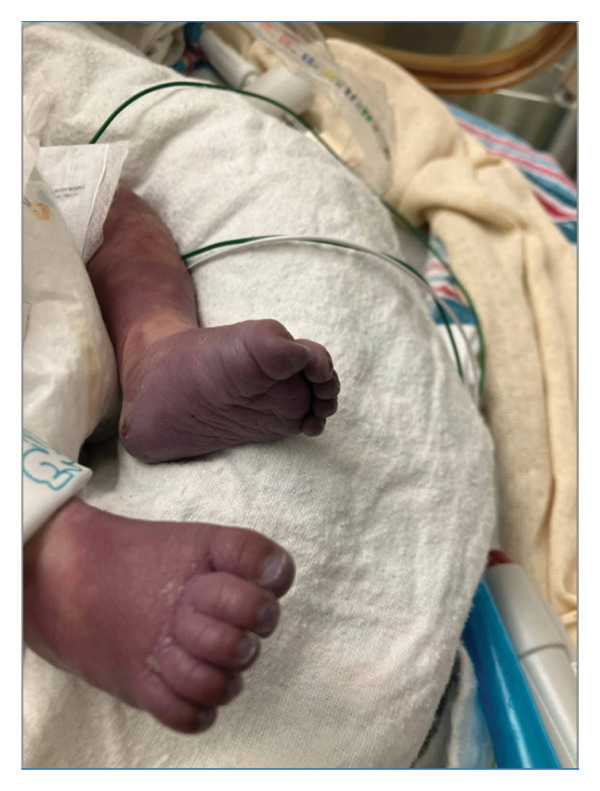
(a)

**Figure:**
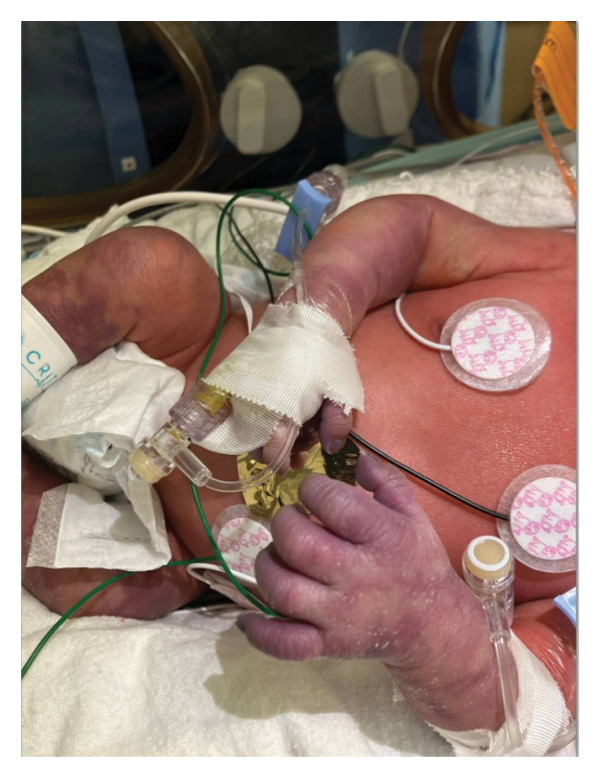
(b)

**Figure:**
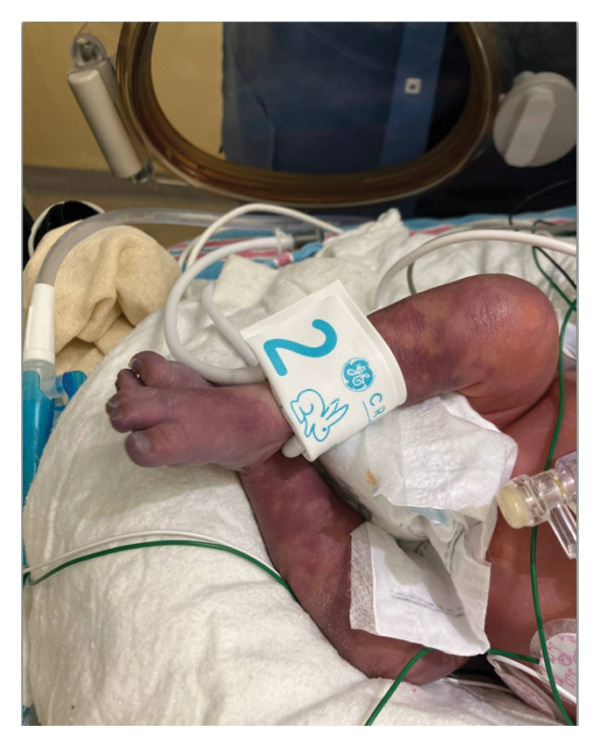
(c)

**Figure:**
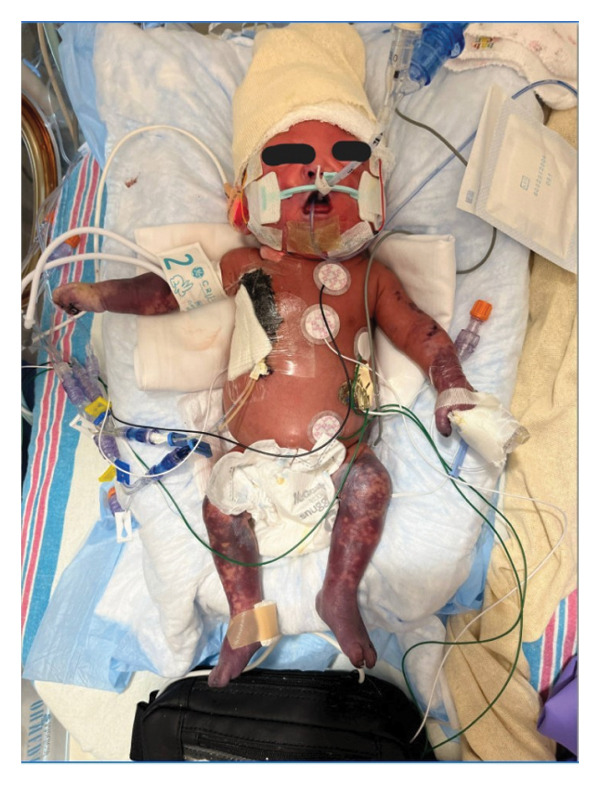
(d)

**Figure:**
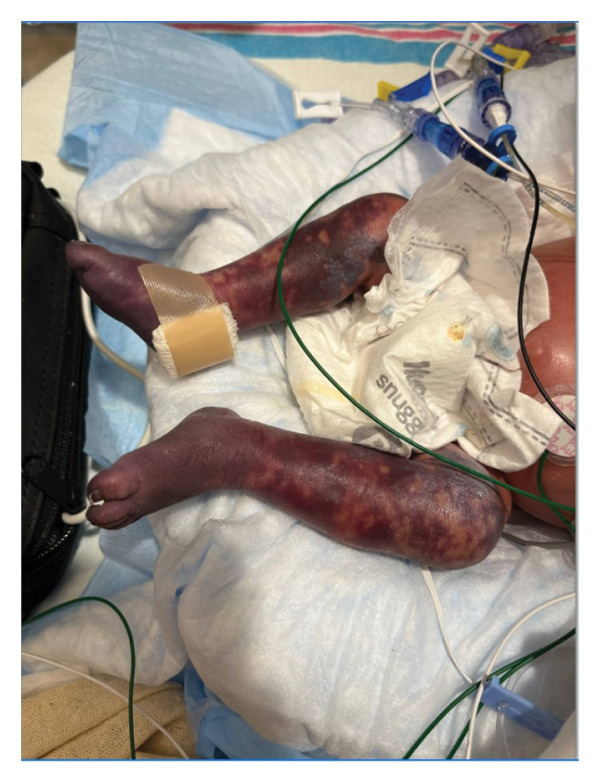
(e)

Figure 2Photos obtained 4 days after the onset of the sepsis showed progressive PF with clearly demarcated cutaneous necrosis involving both hands, feet, forearms, and legs (a, b, c).

**Figure:**
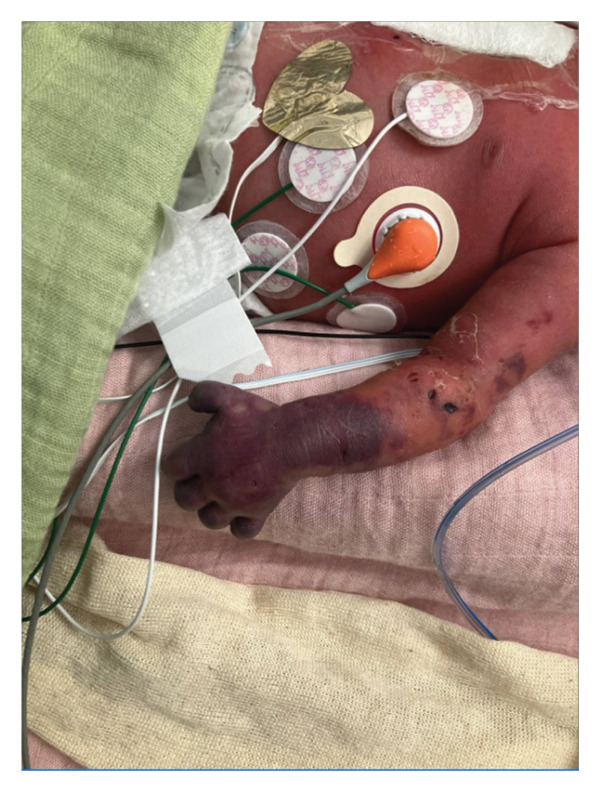
(a)

**Figure:**
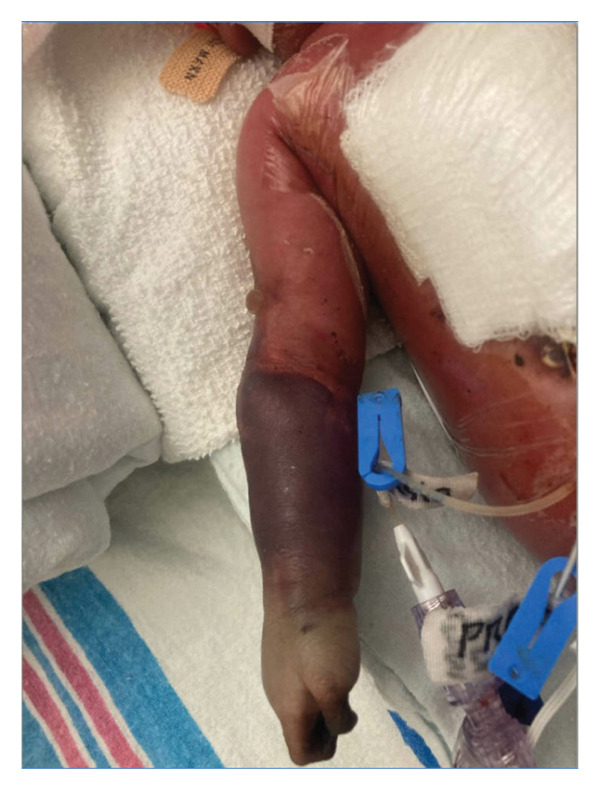
(b)

**Figure:**
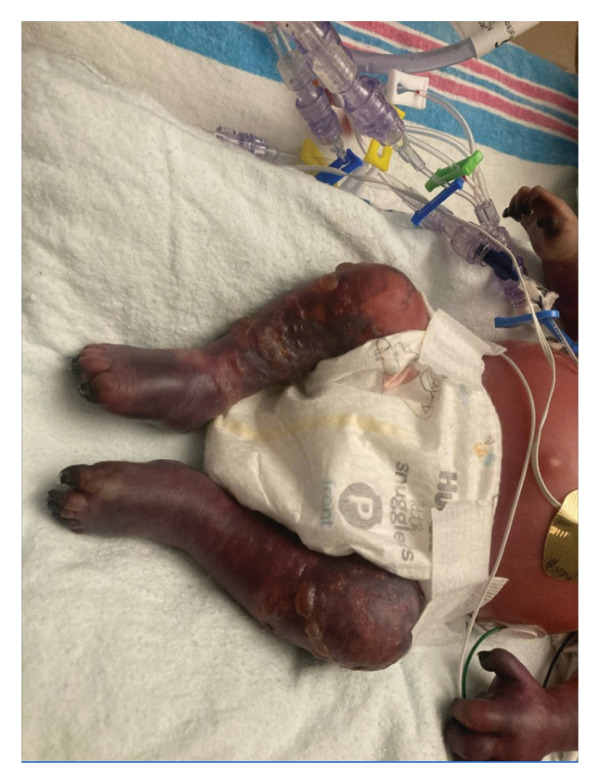
(c)

Figure 4Photos taken 3 weeks after the onset of sepsis showed progressive necrosis of the extremities and autoamputations of the left hands (a), right hand, right distal forearm (b), left foot, left distal leg (c) and right foot (d).

**Figure:**
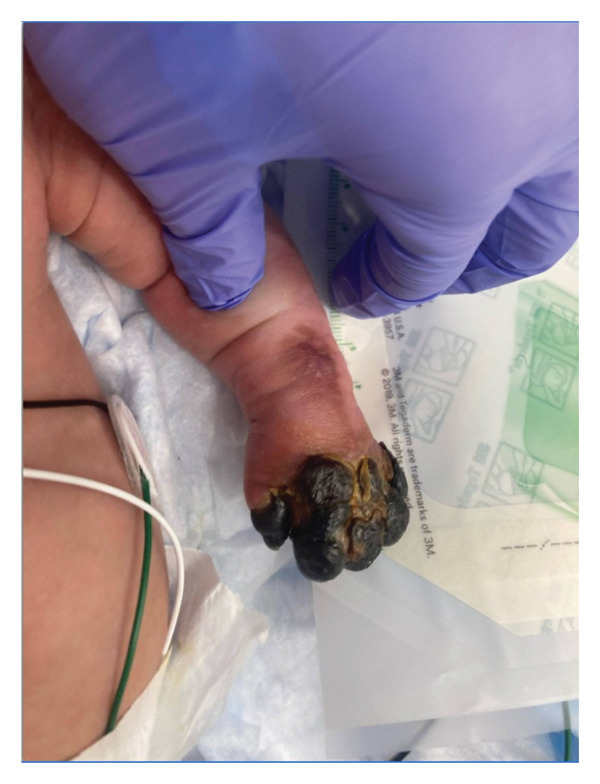
(a)

**Figure:**
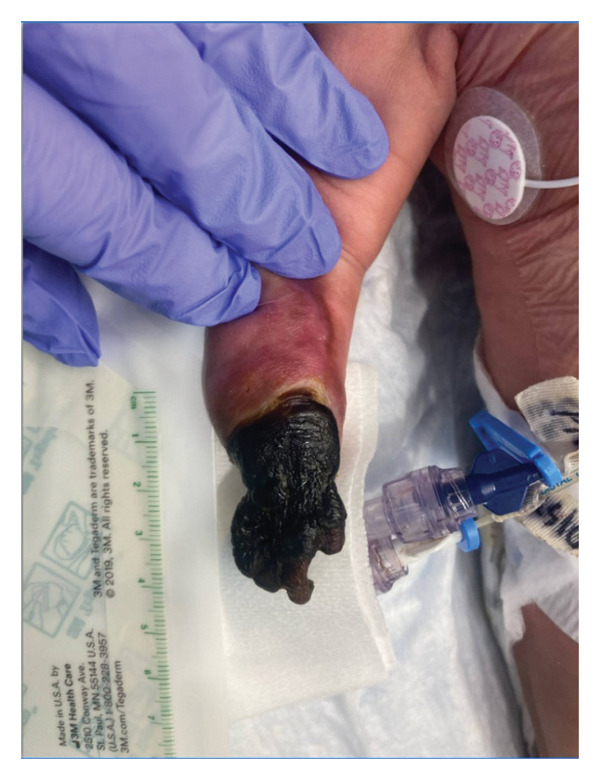
(b)

**Figure:**
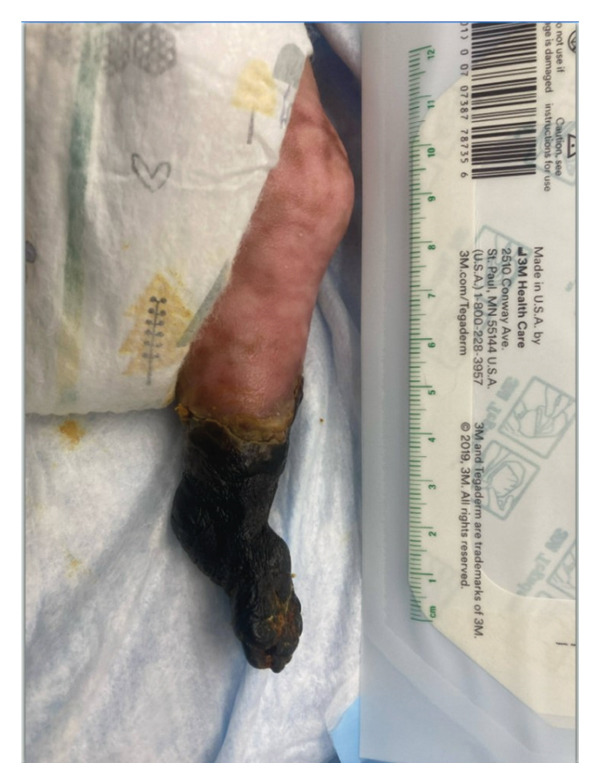
(c)

**Figure:**
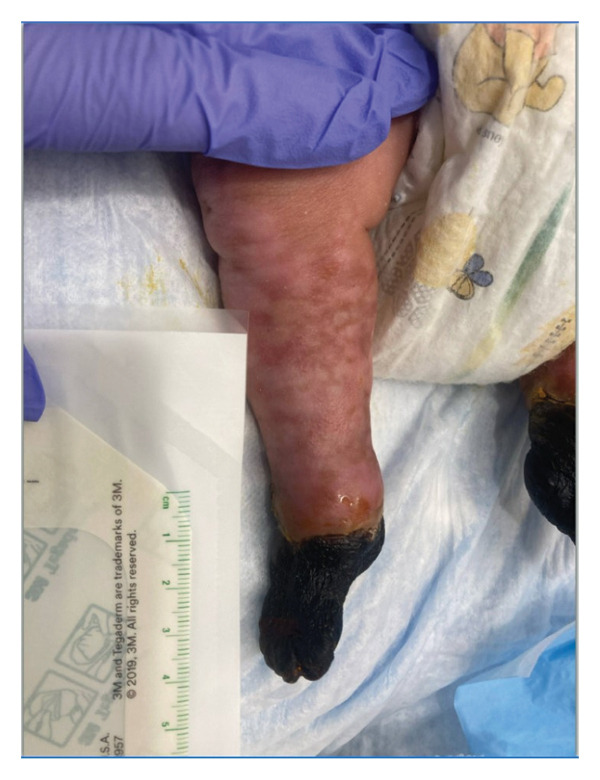
(d)

Figure 5The healed amputated limbs at DOL 220—4 months after onset of sepsis. Shown are the left upper extremity (a), right upper extremity (b), and bilateral lower extremities (c).

**Figure:**
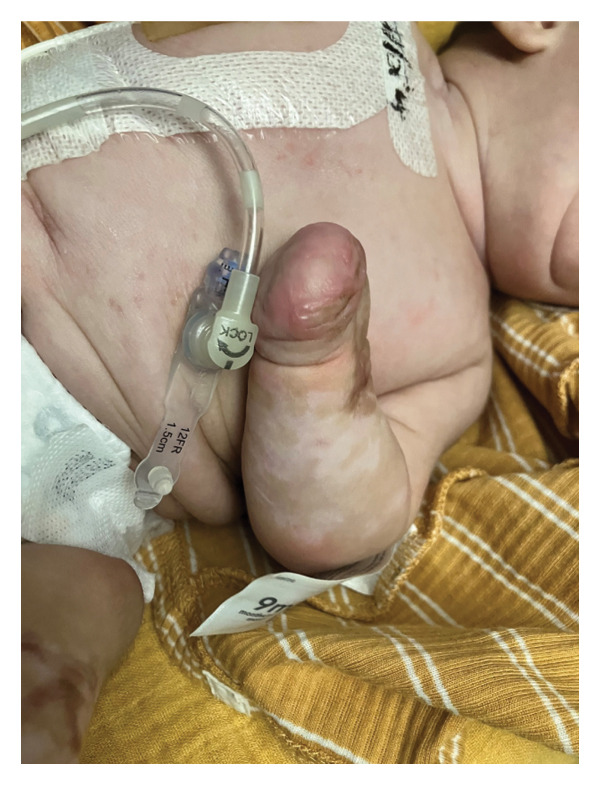
(a)

**Figure:**
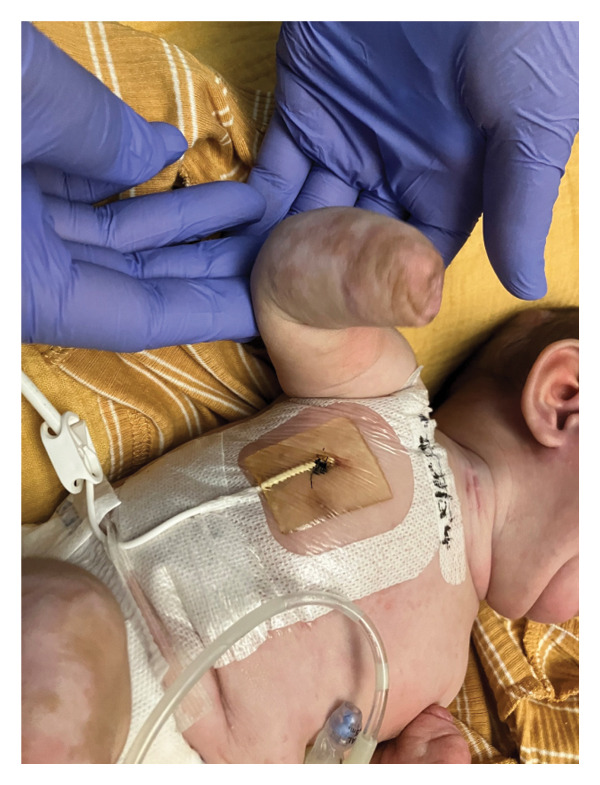
(b)

**Figure:**
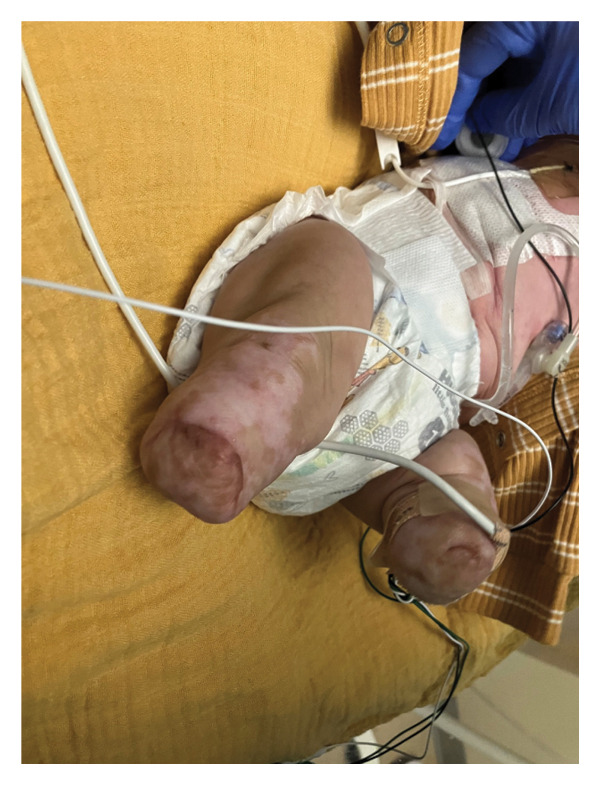
(c)

Figure 6A neurosonogram on DOL 112 (4 days after the onset of sepsis) showed mild ventriculomegaly of lateral ventricles and an echodense area (arrow) in the brain parenchyma (a). Repeat imaging on DOL 146 showed marked dilated lateral and fourth ventricles as well as cystic leukomalacia (b).

**Figure:**
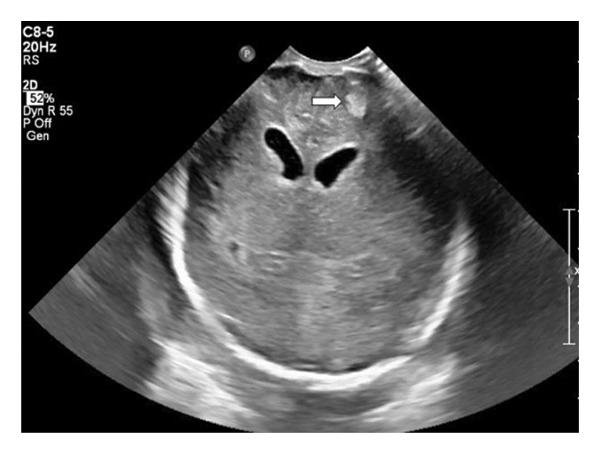
(a)

**Figure:**
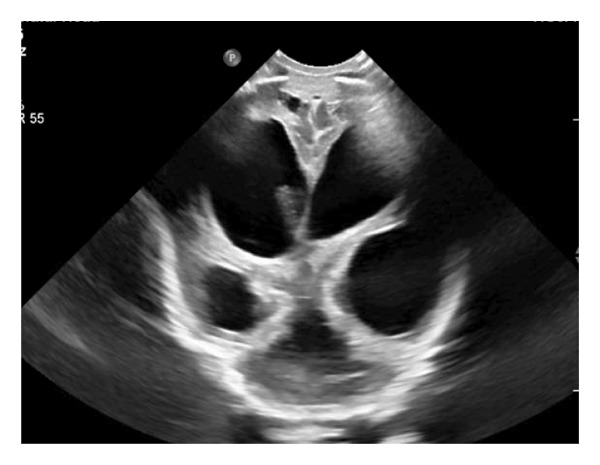
(b)

Figure 7Axial CT images of the head on DOL 222 following ventriculoperitoneal shunt placement) showed marked dilated lateral ventricles, left greater than right, of the lateral ventricles in the coronal (a) and sagittal (b) planes.

**Figure:**
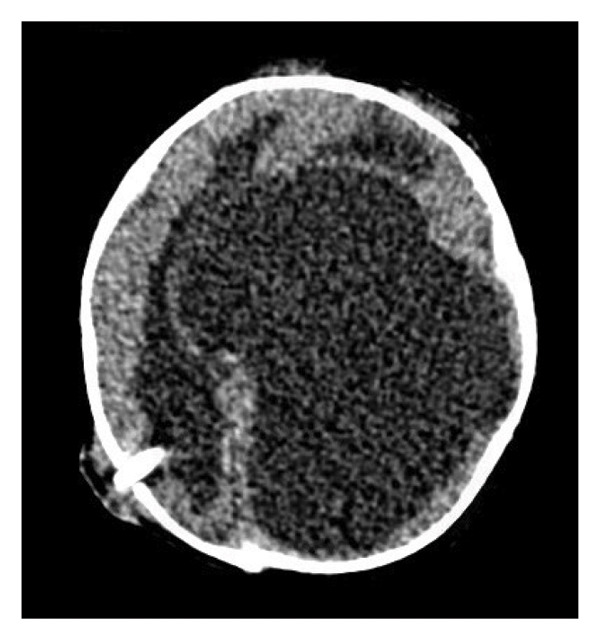
(a)

**Figure:**
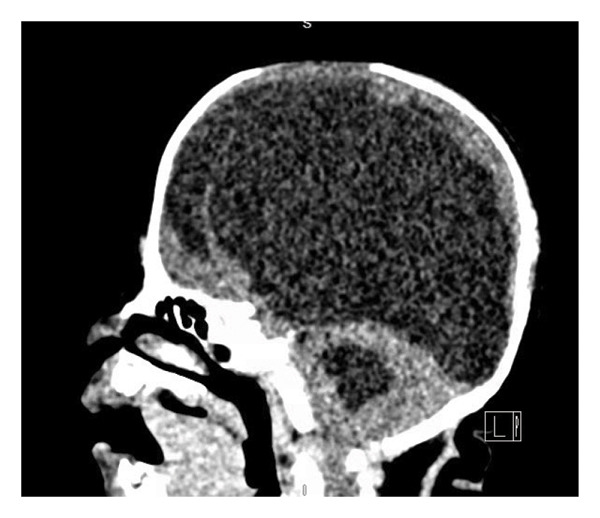
(b)

## 3. Discussion

Very few neonatal cases of GBS‐associated early‐onset sepsis (EOS) and late‐onset sepsis (LOS) progressing to severe PF have been reported. Few groups have described PF in newborns due to early‐onset GBS, and even fewer have reported PF associated with late‐onset GBS [3,5–9] such as our patient. The risk factors for late‐onset GBS sepsis included maternal GBS colonization, young maternal age, preterm birth, HIV exposure, and African ethnicity. Emerging evidence highlights the predominant role of maternal sources in the transmission of late‐onset GBS sepsis. Mucosal colonization may occur perinatally or during the postpartum period, originating from maternal or other sources. GBS‐infected breast milk—with or without associated mastitis—represents a potential route of transmission. Horizontal transmission from nosocomial or community sources is also possible. In the neonatal intensive care unit, nosocomial transmission may be underrecognized, and recurrence of GBS disease has been documented [10]. Our patient’s maternal GBS status was unknown, and the patient was fed with expressed maternal breast milk; these are the possibilities of the maternal sources in the transmission of late‐onset GBS infection in our case.

In earlier reports, patients lost some digits due to PF, but none suffered as extensive a loss of their distal extremities as our patient. In the previously cited case reports, all survivors exhibited poor neurological outcomes, consistent with the course observed in our case (Table 1) [3,5,7–9]. However, this is the first reported case demonstrating extensive involvement of all four limbs due to PF, with detailed documentation of its progression from onset through to surgical amputation of all distal extremities. Additionally, most previously reported neonatal PF cases involved full‐term infants, whereas our case was an extremely premature infant. One group reported PF in a premature infant presumed to be infected but who had negative blood cultures and normal protein C and S levels, whereas another reported PF in a late preterm infant with *Escherichia coli* sepsis [4,12].

**Table 1:** Purpura fulminans associated with GBS.

References	#case	Gender	GA (wk)	AO (day)	EOS/LOS	Meningitis	Outcome	Amputation	Delivery
Isaacman et al. [4]	1	M	Term	16	LOS	Y	Thrombohemorrhagic 3 limbs (LH, RH, RF)	Fingers/toes	V

Lynn et al. [11]	3	M	36	0.3	EOS	Y	Thrombohemorrhagic 2 limbs (lower limbs), porencephalic changes, hemiparesis	Distal extremity scarring	V
		M	36	1	EOS	Y	Thrombohemorrhagic 2 limbs; leukomalacia, optic atrophy	Distal extremity scarring	V
		M	36	1	EOS	Y	Thrombohemorrhagic 4 limbs and scrotum, hydrocephalus	Distal extremity scarring	V

Hon et al. [1]	1	F	35	Birth	EOS	UKN	Thrombohemorrhagic 4 limbs; died at 9 days of life	Death	C/S

Zenciroglu et al. [2]	1	M	39	2	EOS	UKN	Thrombohemorrhagic 4 limbs; visual and hearing impairment	L Fingers, R fingers/toes	V

Albarrak [3]	1	F	Term	2	EOS	Y	Thrombohemorrhagic 4 limbs; died at 4 days of life	Death	V

Elayappen et al. [8]	1	M	27	25	LOS	Y	Thrombohemorrhagic 2 limbs (LH,LF); hydrocephalus	Left fingers/toes	C/S

*Note:* Y, yes; V, vaginal, UKN = unknown.

Abbreviations: AO, age of onset; EOS, early‐onset sepsis; GA, gestational age; LOS, late‐onset sepsis.

The earliest description of PF was by Guelliot in 1884 [13], followed by Henoch in 1886 [14]. According to a 1964 review by Hjort, PF is a disease that affects children. It manifests as skin bleeding that leads to anemia and eventually skin necrosis, and it happens at a varied but distinct latent period after a benign sickness. He speculated that the term would eventually be expanded to include pathologic and laboratory results consistent with consumptive coagulopathy. Our case showed a combination of laboratory tests of DIC: low platelet count and fibrinogen, prolonged PT and PTT, and increased levels of D‐dimer. Although these early descriptions included a clear latent period between the instigating event and the onset of PF, others have suggested two different clinical presentations: (1) an acute form that is immediately superimposed on a bacterial infection and (2) a chronic form that follows viral or idiopathic causes. More recently, homozygous protein C or protein S anticoagulant activity deficits have been linked to a familial type of newborn PF [11]. Here, the consumptive coagulopathy is caused by unchecked extensive thrombus development, which is made possible by several defects. Given the current view that early‐onset GBS syndrome often starts in utero, it may be impossible to identify any latent period, even though the reported individuals seem to reflect the acute variety [6].

In older children and the general population, PF cases are not typically linked to GBS disease. In the reported cases, *Neisseria meningitidis*, *Streptococcus pneumoniae*, *Streptococcus pyogenes*, *Escherichia coli*, *Klebsiella pneumoniae*, and viral infections like varicella have all been implicated [5]. The pathophysiology is not sufficiently detailed to explain the novel role of GBS, despite the response being compared to the antigen‐antibody Arthus reaction or the endotoxin‐induced Shwartzman reaction. PF can be categorized as idiopathic, acquired, or congenital. Infection is the primary cause of acquired PF in neonates, and GBS sepsis is the most prevalent pathogen in this population. Protein C and protein S deficiencies are congenital causes that can cause thrombosis and PF within 72 h of birth. Acute venous thrombosis, warfarin, infections, congenital heart disease, galactosemia, and DIC are examples of acquired causes. Idiopathic PF is rare and typically reported in adolescents [5,11]. Our patient’s late‐onset GBS infection was not prevented by intrapartum GBS chemoprophylaxis, which is consistent with the results of other reports. Protein C and S levels were not checked in our patient, but there was no family history of bleeding or coagulation disorders.

The management of infectious PF includes the use of antibiotics and maintenance of tissue perfusion, which may only be partially helpful. Because this condition is rare and can have life‐threatening symptoms, treatment has been empirical without any control studies being conducted to show efficacy. Therapeutic measures for PF include aggressive cardiorespiratory support, platelet and plasma transfusions, antibiotics, steroids, intravenous immunoglobulin, activated protein C concentrate, heparin, AT III, recombinant tissue plasminogen activator, topical nitroglycerin, epoprostenol, dextran, epsilon‐aminocaproic acid, hormones, and plasmapheresis. In one case report, the infant underwent a double‐volume exchange transfusion to manage persistent acidosis. Other reported interventions include hyperbaric oxygen therapy, heparinization, and epidural sympathetic blockade [2, 4, 14]. However, experience with these treatments in the neonatal population remains limited. Antibiotics were started early in our patient’s illness, but the disease progressed extremely rapidly and caused extensive damage. The infant received a short course of inotropic agents, blood product transfusions, and aggressive cardiopulmonary support. Heparin therapy was contraindicated due to the presence of intracerebral hemorrhage. It has repeatedly been shown to be beneficial when used empirically and is generally recommended in the treatment of PF [4].

Reported outcomes of PF indicate a mortality rate of approximately 50%, primarily due to multiple organ failure commonly associated with the syndrome. Survivors frequently experience significant long‐term morbidity. In three cases of early neonatal PF, all infants survived but demonstrated severely impaired neurological outcomes [1]. Zenciroglu et al. [2] described a 2‐day‐old neonate with extensive PF secondary to GBS septicemia who survived but required amputation and developed cortical blindness along with poor neurological function.

In summary, PF in the neonate is a rare but potentially disabling illness of acute onset associated with high mortality and long‐term morbidity. Our case highlights the rare presentation of severe sequelae due to PF secondary to late‐onset GBS infection in an extremely low birth weight infant. Our patient is the smallest infant reported in the literature with GBS and PF. This is the first case report that documented the extensive evolution of PF secondary to GBS sepsis leading to the amputation of all distal extremities.

## Ethics Statement

Consent was obtained from the parents. The Case Report was not required for approval by the Institute’s Committee on Human Research.

## Conflicts of Interest

The authors declare no conflicts of interest.

## Author Contributions

Surasak Puvabanditsin, Ian Lee, Aline Sandouk, and Amrryn Halari contributed to data gathering and the patient’s diagnosis and treatment.

Surasak Puvabanditsin, Mannan Shah, Su Young Park, and Rajeev Mehta contributed to the drafting and revision of the main manuscript.

## Funding

No funding was received for this manuscript.

## Data Availability

The data that support the findings of this study are available from the corresponding author upon reasonable request.
